# A combination of multimodal physical exercises in real and virtual environments for individuals after chronic stroke: study protocol for a randomized controlled trial

**DOI:** 10.1186/s13063-019-3396-2

**Published:** 2019-07-16

**Authors:** Natalia Araujo Mazzini, Murilo Groschitz Ruas Almeida, José Eduardo Pompeu, Janaine Cunha Polese, Camila Torriani-Pasin

**Affiliations:** 1 Motor Behavior Laboratory, School of Physical Education and Sport, University of São Paulo, São Paulo, SP Brazil; 2 Laboratory of Studies in Technology, Functionality and Aging of the Department of Physical Therapy, Speech and Occupational Therapy, School of Medicine, University of São Paulo, São Paulo, SP Brazil; 3 Department of Physical Therapy, Medical Sciences College of Minas Gerais, Belo Horizonte, MG Brazil

**Keywords:** Stroke, Physical exercises, Virtual reality, Quality of life.

## Abstract

**Background:**

Multimodal physical exercises already have well-established benefits for the post-stroke population that influence gait functional capacity, balance, gait, cognition, and quality of life. This type of intervention can be performed in both real and virtual environments. Considering the characteristics of both environments, it is questioned to what extent the combination of interventions in real and virtual environments could result in improvement in post-stroke impairments.

**Methods/design:**

We will conduct a randomized clinical trial with three groups: a real multimodal group (RMG), a virtual multimodal group (VMG), and a combined multimodal group (CMG). It was estimated that we will need a sample of 36 participants (12 per group). RMG individuals will only perform multimodal physical exercises in a real environment two times per week for 60 min per session for 15 weeks. VMG individuals will perform exercises of the same duration over the same time frame but only in a virtual environment. CMG individuals will hold a weekly session in a real environment and another weekly session in virtual environment. The primary outcome measure will be health-related quality of life, evaluated using the Stroke Impact Scale; effects on cognition (Montreal Cognitive Assessment), balance (Berg Balance Scale), mobility (Timed Up & Go), self-selected gait speed (10-meter walk test), and gait functional capacity (6-min walk test) will be investigated as secondary outcome measures. Participants will be evaluated before the beginning of the intervention, immediately after the end of the intervention, and at 1-month follow-up without exercise. If the data meet the assumptions of the parametric analysis, the results will be evaluated by analysis of variance (3 × 3) for the group factor, with repeated measures while taking into account the time factor. The post hoc Tukey test will be used to detect differences (α = 0.05).

**Discussion:**

This study represents the first clinical trial to include three groups considering physical exercise in real and virtual environments, isolated and combined, that counterbalances the intensity and volume of training in all groups. This study also includes a control of progression in all groups along the 15-week intervention. The outcome measures are innovative because, according to International Classification of Functioning, Disability and Health, activity and participation are the targets for effectiveness evaluation.

**Trial registration:**

Combinação de exercícios físicos multimodais em ambientes real e virtual para indivíduos pós acidente vascular cerebral crônico, RBR-4pt72m. Registered on 29 August 2016.

**Electronic supplementary material:**

The online version of this article (10.1186/s13063-019-3396-2) contains supplementary material, which is available to authorized users.

## Background

The diverse impairments observed after a stroke, associated with the reduction of intrinsic motivation and the presence of preexisting or acquired comorbidities, lead to a vicious cycle of decreased activity and increased exercise intolerance. As a consequence, secondary complications, such as reduced cardiorespiratory fitness, muscle atrophy, osteoporosis, and circulation impairment in the lower extremities, may occur and generate greater dependence in the activities of daily living and impact the social interactions of these individuals [1].

Different modalities of physical exercises already have well-established benefits for individuals after chronic stroke, including repercussions for cardiovascular capacity [2], muscle strength [3, 4], balance [5, 6], gait [7, 8], and cognition [9]. In order to maximize the effects of the exercises, there is a tendency to investigate the effects of multimodal protocols. According to Saunders et al. [10], a multimodal protocol refers to interventions based on the combination of physical exercises of different components, such as cardiorespiratory, muscular strength, and flexibility.

Multimodal physical exercises can be performed in both real and virtual reality environments. The interventions performed in real environments are the most commonly used in the clinical context. Characteristics of interventions performed in real environments include a high interactive relationship between the professional and the patient, high ecological validity, the possibility of individual or group applications, not requiring technological resources, and the ability to be applied in the home according to each patient’s needs.

Conversely, virtual reality-based interventions present features such as an environment rich in visual and auditory information with immediate and multisensory feedback [11], real-time simulation of tasks or environments, three-dimensional interactive and immersive experiences, a computerized interface, active and safe patient participation [12], and the ability to provide information with an external focus of attention [13, 14]. In a systematic review, Laver et al. [15] found that the addition of virtual reality to conventional methods resulted in improved upper limb function. However, they also found insufficient evidence regarding the superiority of virtual reality for promoting walking speed and balance. They were unable to pool results related to cognition, improvement of social participation, and health-related quality of life (HRQoL) because few studies included assessments of cognition and HRQoL to achieve meta-analysis requirements for these outcomes [15]. Therefore, these parameters should be investigated in future studies; in addition, the authors also emphasized the need for training lasting longer than 15 h of intervention and that future studies should set the number of participants screened for eligibility criteria.

Considering the characteristics of both environments, it is questioned to what extent the combination of interventions in the real and virtual environments could result in improvement in post-stroke impairments. There are few studies that have sought to find answers to this question. In the Shin et al. [16] study, the control group performed 1 h of occupational therapy per session, and the experimental group performed 30 min of occupational therapy plus 30 min of virtual reality. The results showed positive effects in both groups, except for the domain related to the limitations due to physical problems measured by the Short Form Health Survey scores, in which experimental group (EG) obtained greater benefits. Rajaratnam et al. [17] found positive results for balance and mobility measurements for the group that performed 40 min of conventional therapy plus 20 min of self-directed virtual reality balance training per session, compared with the control group, which performed 60 min of conventional therapy.

Saposnik and Levin [18] claimed there were few publications regarding the combination of multimodal physical exercises in real and virtual environments. Most of the existing studies did not investigate long-term effects, including follow-up, and added intervention time to the experimental groups, which provided them with an advantage in the total intervention time received. In addition, there is an important diversity in the literature regarding the profile characteristics of individuals with stroke, considering acute, subacute, and chronic patients. Thus, the results found in the previous studies [19–24] do not allow consistent conclusions to be made about the effects of the combination of multimodal exercises in real and virtual environments in individuals after chronic stroke.

This study seeks to answer whether the combination of multimodal physical exercises in real and virtual environments could bring additional benefits to the quality of life, cognition, gait, and balance of individuals after chronic stroke. We also intend to clarify the effects of interventions with multimodal physical exercises when performed only in a real environment or only in a virtual environment and to investigate whether the possible effects remain after 1 month without participating in physical exercises.

This study aims to investigate the effects of a protocol of multimodal physical exercises in real and virtual environments for individuals who have survived a stroke.

## Methods/design

### Trial design

This is a prospective, randomized, single-blind trial of a 15-week exercise program to investigate the effects of the combination of physical exercise in real and virtual environments for chronic post-stroke individuals. It was approved by the Ethics and Research Committee on Human Beings of the School of Physical Education and Sports at the University of São Paulo (CAE no. 40,688,114.9.0000.5391) and is registered with ensaiosclinicos.gov (no. RBR-4pt72m).

**Fig. 1:**
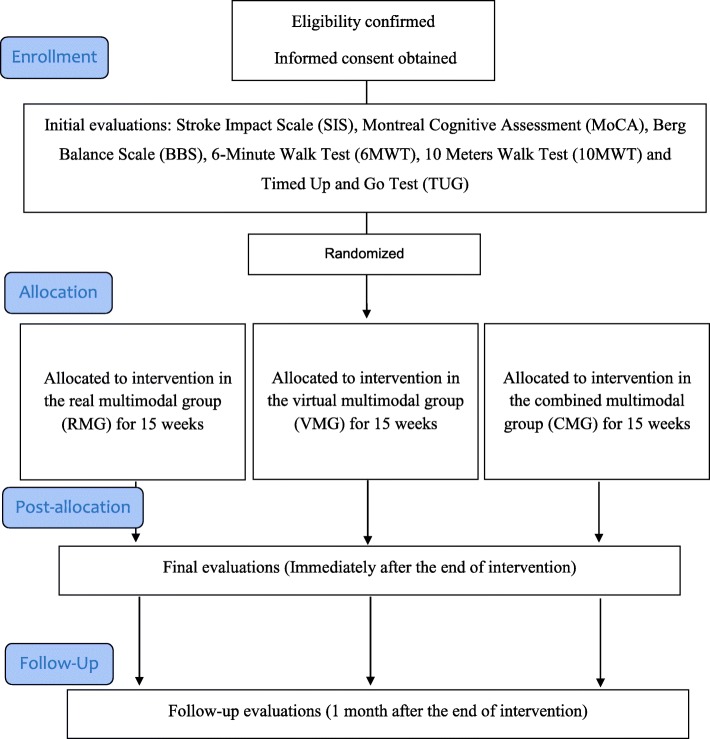
Consolidated Standards of Reporting Trials (CONSORT) flow diagram

All participants and/or their relatives will provide written informed consent prior to participant enrollment. A Consolidated Standards of Reporting Trials (CONSORT) flow diagram of the trial is shown in Fig. 1, and a Standard Protocol Items: Recommendations for Interventional Trials (SPIRIT) checklist is provided in Additional file 1.

### Study setting

A prospective, randomized trial with concealed allocation, blinded assessors, and intention-to-treat analysis will be carried out. The study will be conducted in the Motor Behavior Laboratory in the School of Physical Education and Sports at the University of Sao Paulo, Brazil.

### Eligibility screening

Participants will be recruited from the waiting list for the rehabilitation program at the Physical Education and Sports School in Sao Paulo, Brazil, and will be screened for eligibility by neurological rehabilitation physiotherapy specialists.

### Inclusion and exclusion criteria

Screening will be done to obtain personal data for the individuals (name, sex, date of birth, address, telephone, and schooling), medical history, brain injury topography, and type of locomotion. After this initial screening, the individuals will be referred for a specific medical evaluation in order to investigate their health conditions, request the necessary tests, and follow the medicines used. Only those individuals who receive medical release for the practice of physical exercises will enter the study.

The inclusion criteria will be chronic phase stroke (more than 6 months post-stroke, according to Bernhardt et al. [25]), territory of the lesion in the middle cerebral artery or anterior cerebral artery, both types of stroke (ischemic and hemorrhagic), cognition greater than 24 points on the Mini Mental State Examination, at least 2 months of noninvolvement in other structured forms of physical exercise intervention (community), and no experience in virtual reality games.

The exclusion criteria will be individuals with any type of cardiovascular complication that would contraindicate physical exercise, individuals who underwent surgeries to attenuate clinical conditions resulting from stroke, and those who have undergone chemical blockade to reduce spasticity.

### Informed consent

Individuals who fit the study requirements will receive the informed consent form, which will be duly read and explained by the researcher and signed by the individuals and/or their legal guardians.

### Randomization procedures

Participants who meet the study inclusion criteria will be randomized into one of three sample groups—the real multimodal group (RMG), the virtual multimodal group (VMG), or the combined multimodal group (CMG)—through a draw of numbered (generated by computer) and sealed opaque envelopes. The evaluators have no knowledge of the results of the individual allocation (blinded evaluators). However, the same is impossible to perform with the professionals who will perform the interventions; because the interventions are supervised and progressive, there are clearly distinct protocols between groups of individuals.

To characterize the individuals, the Mini Mental State Examination, Fugl-Meyer Assessment, and Orpington Prognostic Scale will be applied. The individuals will also be characterized by the demographic variables of sex, age, type of stroke, injury time, affected brain hemisphere, and schooling.

### Outcome measures and outcome assessment

The primary outcome will be the domain “activities of daily living” from the Stroke Impact Scale (SIS). This will be used to measure the individuals’ levels of participation. As secondary outcome measures, the other domains from the SIS will be used, as well as their total score and the percentage of recovery from stroke, for the purpose of measuring the range of structures/functions and activities. These will be measured using the Montreal Cognitive Assessment (MoCA), Berg Balance Scale (BBS), 6-minute walk test (6MWT), 10-meter walk test (10MWT), and Timed Up & Go Test (TUG).

All measurements will be carried out by trained evaluators who will not be involved in the interventions and will be blinded to the allocation of individuals to the groups. All individuals will be evaluated at three moments: before the beginning of the interventions (baseline), immediately after the end of the interventions (posttest), and 1 month after the end of the interventions (follow-up). The schedule of enrollment, allocation, and postallocation is provided in Table 1.

**Table 1:** Outcomes assessment time-points and instruments

	Instruments/methods	Enrolment	Baseline	Allocation	Post allocation	
					Post test 15 weeks	Follow up 19 weeks
Eligibility screen: Socio-demographic variables	Structured interview, recording age, brain injury, schooling and medical history	X				
Informed consent	Informed Consent Term	X				
Allocation	3 intervention groups: RMG, VMG or CMG			X		
Quality of life	SIS		X		X	X
Cognitive	MOCA		X		X	X
Gait Functional Capacity	6MWT		X		X	X
Self-selected gait speed	10 MWT		X		X	X
Mobility	TUG		X		X	X
Balance	Berg Balance Scale (BBS)		X		X	X

### Intervention

The intervention for the three sample groups will be 15 weeks long and will consist of two weekly sessions of 60 min each. All interventions will be carried out at the School of Physical Education and Sports of the University of Sao Paulo, Brazil.

The individuals allocated to RMG will carry out sessions in multimodal training in a real environment. The sessions will be divided into four moments, based on guidelines from Billinger et al. [26]: balance and cognition (15 min in duration), training of the aerobic component and muscular strength (20 min each), and a fourth moment focused on flexibility and relaxation (5 min in duration). Aerobic circuits, games, adapted sports, strength training stations, tasks with varying bases of support, double tasks, memorization tasks, and other strategies will be used. Materials will include balls of diverse sizes and constitutions, dumbbells, washers, cones, hula hoops, bladders, wooden sticks, mats, shuttlecocks, and rackets.

Individuals assigned to the VMG group will carry out individualized multimodal training sessions in a virtual environment. For this protocol, there will be eight games of Stability and Balance Learning Environment, a system of virtual reality developed by Motekforce Medical (Amsterdam, the Netherlands), which is composed of a 10.76-ft^2^ force platform, three projectors, six infrared cameras, a sound system, and a touchscreen panel for selection and control of variables. The games of this virtual reality device were produced and adapted to be beneficial for special populations that have balance and movement disorders, such as people with neurological disorders (stroke, cerebral palsy, Parkinson’s disease, traumatic brain injury), orthopedic disorders (amputees, osteoarthritis, musculoskeletal disorders), and the geriatric population (elderly with increased risk of falling). Stability and Balance Learning Environment requires the player to move the entire body, which includes tasks of reach, stationary gait, and others.

**Fig. 2:**
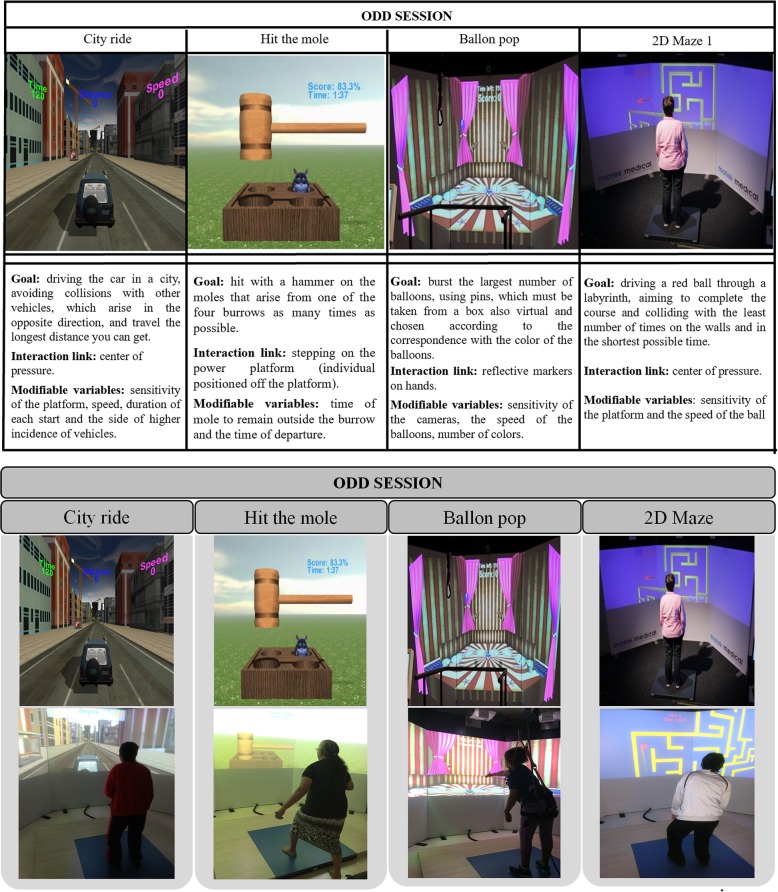
Virtual reality games selected for the odd-game sessions and individuals’ practice

**Fig. 3:**
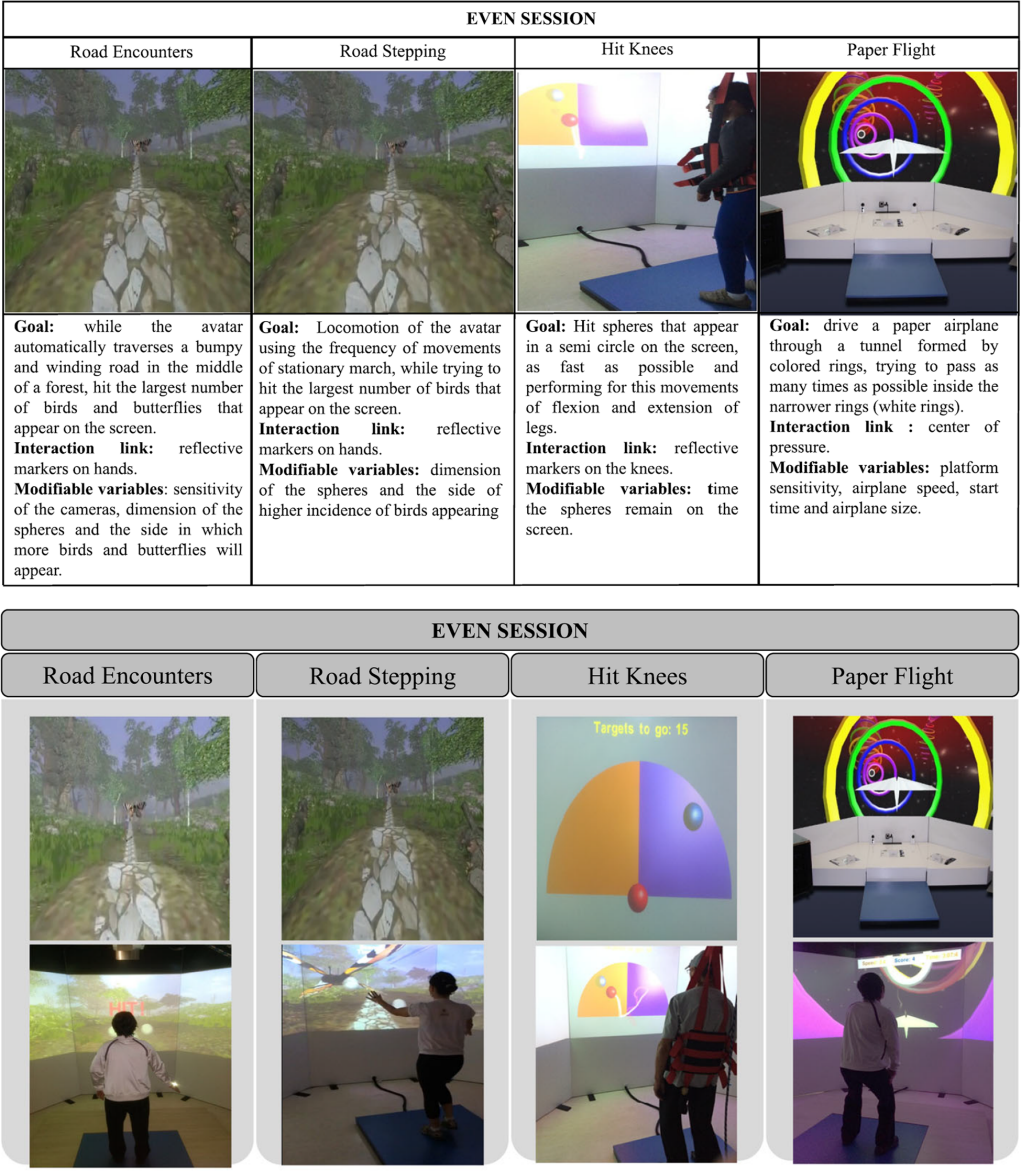
Virtual reality games selected for the even-game sessions and individuals’ practice

These games were grouped into two blocks of sessions that will be applied alternately throughout the intervention. The odd sessions (Fig. 2) will include the games balloon pop, city ride, hit the mole, and 2d maze, and the even sessions (Fig. 3) will consist of the games road encounters, road stepping, paper flight, and hit knees. The number of selected games as the group session in two models aims to make the most motivating interventions possible and keep the characteristics between the games of both models similar.

In order to guarantee the multimodal character of the virtual intervention and to make it similar to the real intervention, the games were classified according to the perceptual and motor demands of the virtual reality systems protocol developed by Cairolli et al. [27]. The use of this classification protocol ensured that the games placed similar demands on the individuals. For example, the demand for strength and cardiovascular resistance could be consistent across the selected games.

Finally, individuals in the CMG group will perform an intervention that combines the protocols of the two other groups described above. In this way, one of the weekly sessions will be held in a real environment and the other in a virtual environment.

Before the start of all sessions, regardless of the group, the individuals’ blood pressure will be verified in order to ensure that no one initiates the practice of physical exercise with a blood pressure greater than 160/105 mmHg [28]. This contributes to the safety of the practice. To register and control the data, stethoscopes (Efficacy line, Bunzl Saúde, Brazil) and manual blood pressure cuff aneroid sphygmomanometers (Solidor, Bunzl Saúde, Brazil) will be used.

Aerobic exercise intensity control will be performed to ensure the safety of the interventions in the three groups. During the interventions, the individuals’ heart rate (HR) will be monitored constantly in order to keep it aligned with the range prescribed individually using the Karvonen formula ((HR_maximum_ − HR_rest_) × percentage HR intensity + HR_rest_) [29]. We will use 40% and 70% of the reserve HR, as determined by Billinger et al. [26], as the lower and upper intensity thresholds, respectively. The maximum HR will be calculated using the formula 220 − age in years [30], except in individuals who use beta-blockers. Thus, the maximum HR will be calculated by the formula 164 − 0.7 × age in years [31]. For the control of HR ranges, we will use Polar brand H7 frequency meters synchronized with the Polar Team application (Polar, Bethpage, NY, USA). As a complementary form to intensity control, we will use the Borg Rating of Perceived Exertion [32, 33], which correlates with the objective measures of the workload level and HR [24]. The individuals’ perception of effort will be verified before the beginning of each session and soon after the onset of the aerobic component in the real environments, or soon after the practice of each of the games in the virtual environment sessions. We will adjust the offer of stimuli that indicate perceptions between values 11 and 14, as determined by Billinger et al. [26].

Resistance exercise intensity will also be controlled according to Billinger et al. [26]. All individuals will perform eight to ten exercises, preferably involving the major muscle groups. Each exercise will include 1 to 3 sets of 10 to 15 repetitions.

Every 5 weeks, the aerobic and resistance exercise intensity will be adjusted in terms of progression. By the end of the 15-week intervention period, we will have performed two progressions. The aerobic component will include gradual increases (10%) of the HR percentage, and the resistance component will increase through larger numbers of sets or repetitions for each exercise.

### Measurements

*Quality of life* will be evaluated through the SIS. This scale evaluates quality of life by measuring 59 items categorized in 8 domains: strength (4 items), hand function (5 items), activities of daily living/instrumental activity of daily living (10 items), mobility (9 items), communication items [7], emotion (9 items), memory and reasoning (7 items), and participation function (8 items). Each item is rated on a 5-point Likert scale in terms of difficulty the patient has experienced in completing each item. Summative scores will be generated for each domain, ranging from 0 to 100. An extra question will be asked in order to know, on a scale from 0 to 100, how much the individual feels they have recovered since their stroke. Our primary outcome measure will be the domain of the activities of daily living/instrumental activity of daily living, and the rest of the domains will be used as secondary outcome measures [34]. The Brazilian version of SIS 3.0 has satisfactory internal consistency, test-retest reliability, convergent validity, and discriminant validity in stroke patients [34]. SIS 3.0 is a specific measure of psychometrically robust HRQoL that can be useful in assessing the consequences of stroke in different cultural contexts [34]. For some of the domains that make up this scale, there are values of minimum detectable change (MDC) and clinically important difference (CID). According to Lin et al. [35], the values are strength (MDC 24.0 and CID 9.2), activities of daily living/instrumental activities of daily living (MDC 17.3 and CID 5.9), mobility (MDC 15.1 and CID 4.5), and hand function (MDC 25.9 and CID17.8).

*Cognition* will be evaluated through the MoCA. MoCA was developed as an instrument of rapid screening for mild cognitive dysfunction and for evaluating different cognitive domains, such as attention and concentration, executive functions, memory, language, constructional visual skills, conceptual thinking, calculations, and logical reasoning. The scale ranges from 0 to 30 [36]. In the chronic phase of stroke, MoCA has a good correlation with other short cognitive tests and shows high sensitivity and specificity in the prediction of post-stroke cognitive deterioration [37]. There is no consensus on the cutoff to define cognitive impairment for stroke, but most authors use a 26-point cutoff. There are no MDC values for this scale [38]. For the subacute phase of stroke, the scale shows excellent internal consistency (α = 0.78) [39].

*Balance* will be evaluated through the BBS, which organizes the quantitative description (classification of 0 to 4) of functional balance ability into 14 items common to daily life. It has a minimum score of 0 and a maximum of 56 [40]. The measurement’s MDC is 4.66 [41], and it has excellent test-retest reliability (intraclass correlation [ICC] = 0.98) [42].

*Gait functional capacity* will be evaluated through the 6MWT, which quantifies the maximum distance, expressed in meters, that the individual is able to walk in 6 min [43]. The measurement’s MDC is 36.6 m [44]; the CID is 34.4 m [45]; and it has excellent test-retest reliability (ICC = 0.99) [44].

*Self-selected gait speed* will be evaluated through the 10MWT, which determines the average speed, expressed in meters per second, that the individual applies for a distance of 10 m. The measurement CID is 0.06 m/s [46], and it has excellent test-retest reliability (ICC = 0.94) [44].

*Mobility* will be evaluated through the TUG, which determines the time, expressed in seconds, that the individual takes to perform a task involving a change of direction, transfer, and gait [47]. The measurement’s MDC is 2.9 s, and it has excellent test-retest reliability (ICC = 0.96) [44].

### Statistical analysis

It was estimated that a sample of 36 participants (12 per group) would provide 88% power (α = 5%) to detect a difference between group means of 17.3 (MDC of the activities of daily living/instrumental activities of daily living domain of the SIS) using the program G*Power 3 [48], which takes into account the number of sample groups and the number of evaluation measures. To reach this total number of individuals, at least 43 individuals must be recruited, allowing a dropout rate of 20%.

For the mathematical treatment and statistical analysis of the data, Excel software (Microsoft, Redmond, WA, USA) will be used for the tabulation of data, and SPSS version 13 software (SPSS, Chicago, IL, USA) will be used for statistical analyses. A significance level of 5% will be adopted. Initially, for the characterization of the sample, descriptive analysis and the *t* test will be used to examine differences between the RMG, VMG, and CMG groups. Subsequently, for the analysis of the normality and homogeneity of the results of the primary outcome measure and secondary outcomes, the Shapiro-Wilk and Levene tests will be used, respectively. After observing the assumptions for normality and homogeneity, parametric analysis will be performed using two-way analysis of variance (3 groups by 3 moments [baseline, post-test, and follow-up]) and post hoc Tukey test. The mean between-group difference and 95% confidence intervals will be reported for all outcomes. If any subject gives up the protocol, their measured outcomes will be analyzed on an intention-to-treat basis.

## Discussion

The proposed clinical trial is of high clinical significance to the field of neurological rehabilitation and to stroke in particular. Physical exercises are an important intervention strategy to promote improvement of gait functional capacity, muscle strength, balance, gait, and cognition in individuals after stroke [9]. In addition, physical exercises improve the quality of life for the post-stroke population. Billinger et al. [26] argued that physical exercise modalities should complement each other to provide a more integrated form of intervention. Thus, multimodal physical exercise protocols must be implemented for this population.

However, the effects of multimodal physical exercises when performed in different environments are not well established in the literature, and the effects of the combination of real and virtual environments are not known. Thus, the findings of this clinical trial may clarify such shortcomings, especially regarding outcomes related to social participation and activity, components of the International Classification of Functioning, Disability and Health that have been poorly investigated.

Currently, healthcare professionals are advised to offer broad, nonspecific recommendations regarding exercise. This new information on the effectiveness of physical exercise in a real or virtual environment will allow health professionals to make evidence-based treatment recommendations for the best environment in which patients can carry out their physical exercise prescription.

The design of the proposed study has strong points, such as the presence of three sample groups that are similar in frequency, intensity, and volume of training; a period of intervention longer than that usually found in currently published studies; an assessment of outcomes in follow-up; and an analysis of evaluation measures in the context of social participation, one of the domains of the International Classification of Functioning, Disability and Health.

In conclusion, this study represents the first clinical trial to include three groups while considering the prescription of physical exercise in real and virtual environments, both isolated and combined, that counterbalances the intensity and volume of the training in all groups. This study also includes the control of progression in all groups during the 15-week intervention. Although very revealing to evaluate the effectiveness of an intervention, measures evaluating the domains of activity and participation are rarely investigated in the literature. Thus, our study is innovative because it includes these measures, differing from the vast majority of studies, which evaluate only aspects related to body structures and functions.

In addition, after the data analysis, we intend to perform a cost-benefit review, based on Lloréns et al. [49]. This analysis will include human and material resources as well as professional-patient relationships. This analysis will include the comparison between our study and that of Saposnik et al. [50], which showed low-cost and easy-to-access interventions being as useful as the least accessible and least cost-effective.

## Trial status

This study protocol is in progress and is not yet related to requests.

Date recruitment began on August 30, 2016.

Approximate date when recruitment will be completed: first half of 2019.

Date of protocol registration: August 25, 2016.

Protocol version number: RBR-4pt72m. Proof review: 27th May 2019.

## Additional file

Additional file 1: SPIRIT 2013 checklist: recommended items to address in a clinical trial protocol and related documents. (PDF 116 mb)

## Data Availability

The datasets used and/or analyzed during the current study are available from the corresponding author on reasonable request.

## Abbreviations

10MWT: 10-Meter walk test
6MWT: 6-Min walk test
BBS: Berg Balance Scale
CID: Clinically important difference
CMG: Combined multimodal group
HR: Heart rate
HRQoL: Health-related quality of life
ICC: Intraclass correlation
MDC: Minimum detectable change
MoCA: Montreal Cognitive Assessment
RMG: Real multimodal group
SIS: Stroke Impact Scale
TUG: Timed Up & Go Test
VMG: Virtual multimodal group
